# Associations of foot and ankle characteristics with knee symptoms and function in individuals with patellofemoral osteoarthritis

**DOI:** 10.1186/s13047-020-00426-8

**Published:** 2020-09-23

**Authors:** Jade M. Tan, Kay M. Crossley, Shannon E. Munteanu, Natalie J. Collins, Harvi F. Hart, Joel W. Donnar, Gearoid Cleary, Isobel C. O’Sullivan, Liam R. Maclachlan, Catherine L. Derham, Hylton B. Menz

**Affiliations:** 1 Discipline of Podiatry, School of Allied Health, Human Services and Sport, La Trobe University, Melbourne, 3086 Australia; 2 La Trobe Sport and Exercise Medicine Research Centre, School of Allied Health, Human Services and Sport, La Trobe University, Melbourne, 3086 Australia; 3 School of Health and Rehabilitation Sciences, The University of Queensland, Brisbane, 4072 Australia; 4 Department of Physical Therapy, Faculty of Health Sciences, Collaborative Training Program in Musculoskeletal Health Research, and Bone and Joint Institute, Western University, London, N6A 3K7 Canada

**Keywords:** Patellofemoral, Osteoarthritis, Foot, Pain, Function

## Abstract

**Background:**

Foot and ankle characteristics are associated with patellofemoral pain (PFP) and may also relate to patellofemoral osteoarthritis (PFOA). A greater understanding of these characteristics and PFOA, could help to identify effective targeted treatments.

**Objectives:**

To determine whether foot and ankle characteristics are associated with knee symptoms and function in individuals with PFOA.

**Methods:**

For this cross-sectional study we measured weightbearing ankle dorsiflexion range of motion, foot posture (via the Foot Posture Index [FPI]), and midfoot mobility (via the Foot Measurement Platform), and obtained patient-reported outcomes for knee symptoms and function (100 mm visual analogue scales, Anterior Knee Pain Scale [AKPS], Knee injury and Osteoarthritis Outcome Score, repeated single step-ups and double-leg sit-to-stand to knee pain onset). Pearson’s r with significance set at *p* < 0.05 was used to determine the association between foot and ankle charateristics, with knee symptoms and function, adjusting for age.

**Results:**

188 participants (126 [67%] women, mean [SD] age of 59.9 [7.1] years, BMI 29.3 [5.6] kg/m^2^) with symptomatic PFOA were included in this study. Lower weightbearing ankle dorsiflexion range of motion had a small significant association with higher average knee pain (partial r = − 0.272, *p* < 0.001) and maximum knee pain during stair ambulation (partial r = − 0.164, *p* = 0.028), and lower scores on the AKPS (indicative of greater disability; partial r = 0.151, *p* = 0.042). Higher FPI scores (indicating a more pronated foot posture) and greater midfoot mobility (foot mobility magnitude) were significantly associated with fewer repeated single step-ups (partial r = − 0.181, *p* = 0.023 and partial r = − 0.197, *p* = 0.009, respectively) and double-leg sit-to-stands (partial r = − 0.202, *p* = 0.022 and partial r = − 0.169, *p* = 0.045, respectively) to knee pain onset, although the magnitude of these relationships was small. The amount of variance in knee pain and disability explained by the foot and ankle characteristics was small (R^2^-squared 2 to 8%).

**Conclusions:**

Lower weightbearing ankle dorsiflexion range of motion, a more pronated foot posture, and greater midfoot mobility demonstrated small associations with worse knee pain and greater disability in individuals with PFOA. Given the small magnitude of these relationships, it is unlikely that interventions aimed solely at addressing foot and ankle mobility will have substantial effects on knee symptoms and function in this population.

**Trial registration:**

The RCT was prospectively registered on 15 March 2017 with the Australia and New Zealand Clinical Trials Registry (ANZCTRN12617000385347).

## Background

Patellofemoral osteoarthritis (PFOA) is a burdensome condition that is present in up to 40% of individuals with knee pain [1]. Compared to the more widely investigated tibiofemoral osteoarthritis (TFOA), PFOA has a higher incidence in individuals aged 50 years and above [2], results in more pain, stiffness, and functional impairments [3, 4], and can affect individuals as young as 26 [5–7]. The pain that accompanies PFOA is associated with physical activity limitations [8] and reduced quality of life [9], and, therefore, a likely reduction in occupational capacity.

PFOA shares some common impairments with patellofemoral pain (PFP), with evidence also suggesting that PFP in younger adults may be related to PFOA [5, 10–12]. Pronated foot posture (measured via the Foot Posture Index) [13], earlier peak rearfoot eversion [14], greater navicular drop [15], and reduced weightbearing ankle dorsiflexion range [16], are foot and ankle characteristics which have been associated with the presence [13, 14, 16] and development [15] of PFP. Considering the biomechanical link between the foot and lower limb joints higher up the kinetic chain [10, 17–19], and the similarities between PFP and PFOA, it is possible that certain foot and ankle characteristics may also be impaired [10] and therefore influence knee symptoms and function in individuals with PFOA.

Little is known about foot and ankle characteristics in people with PFOA, with limited evidence from one study indicating that individuals with PFOA (*n* = 51) have less ankle dorsiflexion range and greater midfoot mobility compared with heathy controls (*n* = 23) [20]. The relationship of foot and ankle characteristics with knee symptoms and function, which may assist clinicians in making informed treatment decisions to improve symptoms and function associated with PFOA, is not known. For instance, knowledge of foot and ankle characteristics in other knee conditions such as PFP, are known to influence treatment decisions regarding in-shoe interventions such as foot orthoses [21, 22]. Therefore, the objective of this study was to explore cross-sectional relationships between weightbearing ankle dorsiflexion range of motion, foot posture, and midfoot mobility with knee symptoms and function in individuals with clinically diagnosed predominant PFOA.

## Methods

This cross-sectional exploratory study used baseline data obtained from 188 participants with clinically diagnosed PFOA who were recruited for a randomised controlled trial (RCT) investigating the efficacy of foot orthoses. The study protocol has been described in detail [23]. The study was approved by the La Trobe University (HEC16–113) and The University of Queensland (2017000284) human ethics committees, and prospectively registered with the Australia and New Zealand Clinical Trials Registry (ANZCTRN12617000385347). All participants gave written informed consent prior to study enrolment.

### Participants

Participant recruitment occurred between January 2017 and January 2019. Several sources were utilised, including free and paid print and digital advertising, stands at local markets, referrals from orthopaedic hospital outpatient departments and practitioners involved in the study, and mail-outs to patients of the La Trobe University Health Sciences Clinic. Volunteers who responded to advertisements underwent a two-stage screening process. Firstly, an emailed questionnaire or telephone interview screened for key exclusion criteria. Potentially eligible volunteers were then invited to attend a comprehensive physical screening appointment conducted by a physiotherapist or podiatrist with a minimum of 5 years of musculoskeletal experience, to confirm eligibility.

The inclusion criteria for the RCT used a clinical diagnosis of PFOA [4] adapted from the National Institute for Health and Care Excellence (NICE) guidelines [24], which consisted of the following: (i) aged 50 years and over; (ii) anterior or retropatellar knee pain aggravated by at least two activities that load the patellofemoral (PF) joint (e.g. stair ambulation, squatting, rising from sitting); (iii) pain during these activities on most days in the previous month; (iv) pain severity during aggravating activities of at least ≥3 on an 11-point numerical rating scale; (v) symptoms present for at least 3 months; and (vi) no morning joint-related stiffness that lasted longer than 30 min. If bilateral PFP was reported, only the most symptomatic side was included [25].

Exclusion criteria were: (i) predominant knee pain from other knee structures (e.g. TFJ), hip, or lumbar spine; (ii) use of any shoe inserts or knee injections within the previous 3 months; (iii) commencement of new physical therapy treatment for PFP (e.g. new intervention, or modifications to an existing intervention such as therapeutic exercise) within the previous 3 months; (iv) any foot pain or foot condition precluding the use of foot orthoses or flat shoe inserts; (v) a history of major reconstructive lower limb surgery (e.g. anterior cruciate ligament reconstruction, osteotomy, or arthroplasty); vi) planned lower limb surgery in the following 12 months; (vii) any neurological or systemic inflammatory arthritis disorder; viii) major medical conditions (e.g. cancer); (ix) contraindications to x-ray (e.g. pregnancy, breastfeeding); and (x) an inability to understand written and spoken English.

### Outcome measures

#### Participant characteristics

(i) Anthropometric measures were collected from each participant, including height, body mass, and waist circumference, and body mass index (BMI) was calculated.
(ii) Pain duration recorded in years, ascertained via an open-ended question, “*How long have you had your knee pain?*”.
(iii) History of knee injury on the study limb ascertained via an open-ended question, “*Have you injured your symptomatic knee in the past?*”.
(iv) History of knee surgery on the study limb ascertained via an open-ended question, “*Have you had surgery to your symptomatic knee in the past?*”.

#### Foot and ankle measures

The following valid and reliable foot and ankle measures were performed.

**Fig. 1:**
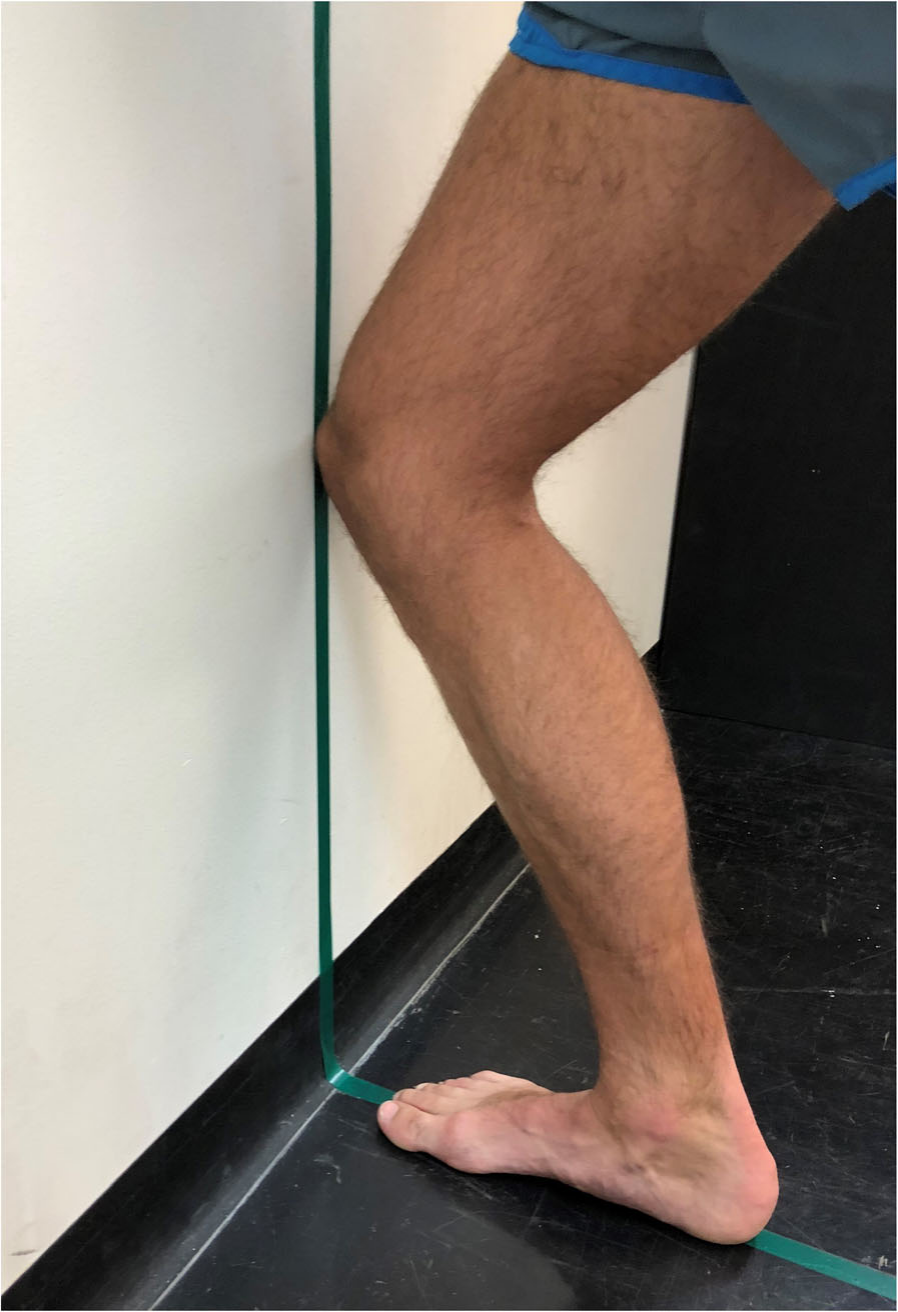
Weightbearing ankle dorsiflexion range

(i) Weightbearing ankle dorsiflexion range of motion was measured using a weightbearing lunge test (see Fig. 1) [26]. A line of tape was placed on the floor perpendicular to the wall (horizontal line) and continued vertically up the wall. This was to ensure the plane of movement was consistent for all participants. Participants were instructed to place the midpoint of their calcaneus and second toe on the horizontal line and lunge forward with a flexed knee, so their knee touched the wall, ensuring the foot remained plantigrade. Participants gradually moved their foot away from the wall to the furthest point where the foot remained plantigrade and the knee was still touching the wall. The distance from the end of the longest toe to the wall was then measured (centimetres). This was repeated three times and an average score calculated [27].
(i) Foot posture was quantified using the Foot Posture Index – 6 items (FPI-6) [28]. Participants stood in a relaxed bipedal stance position, and six observations (talar head palpation, supra- and infra- lateral malleolar curvature, prominence of the talonavicular joint, congruence of the medial longitudinal arch, abduction/adduction of the forefoot, and inversion/eversion of the calcaneus) were documented. Each of the six items was awarded a score ranging from − 2 to + 2, with the six items summated to produce a final score ranging from − 12 to + 12. Higher scores represent a more pronated foot posture [29].
(ii) Midfoot mobility was measured using the Foot Measurement Platform [30]. This clinical assessment tool measures the vertical height and medio-lateral width of the midfoot during weightbearing and non-weightbearing. Participants stood in a relaxed bipedal stance position whilst midfoot arch height and midfoot width measurements were taken. These measurements were then repeated whilst the participant was seated in a non-weightbearing position, with the knee flexed at 90 degrees. All weightbearing and non-weightbearing measures were taken at 50% of the total foot length. The difference in midfoot arch height and midfoot width between weightbearing and non-weightbearing was then determined, and midfoot mobility magnitude was calculated in millimetres (√[midfoot height mobility^2^ + midfoot width mobility^2^]), with a larger score indicating greater midfoot mobility [31].

#### Measures of knee symptoms and function

Knee symptoms and function measures were chosen based on previous research demonstrating validity and reliability of these measures in PFP [32–35] and PFOA [33, 36] populations, and ease of application within the clinical setting.

##### Knee pain provocation tests

(i) Number of repeated single step-ups to knee pain onset: participants started with both feet on a bottom step and were asked to ascend a single set of steps, leading with their nominated study limb, and finishing the ascent with their non-study limb. Participants return to the starting position (leading with their non-study limb) and repeated this until either the first onset of PFOA pain was felt, or the first increase in pain (if pain was constant), or a maximum of 30 repetitions was achieved.
(ii) Number of repeated double-leg sit-to-stands to knee pain onset: participants were asked to rise from a seated position, keeping both feet firmly on the ground and refraining from using any forward momentum to rise up out of the chair, and return to their starting position. Cadence was set at two seconds per rise and lowering. This was repeated continuously until either the first onset of PFOA pain was felt, the first increase in pain (if pain was constant), or a maximum of 30 repetitions was achieved.

##### Patient-reported measures of knee symptoms and function

(i) Severity of knee pain over the past week was measured using five 100 mm visual analogue scales (VAS), with 0 mm representing ‘no pain’, and 100 mm representing ‘worst pain possible’ [32]. The five VAS used were as follows: average pain, worst pain, maximum pain during stair ambulation, maximum pain when squatting, and maximum pain when rising from sitting.
(ii) The Anterior Knee Pain Scale (AKPS) [34] is a questionnaire which consists of 13 categories with items related to limping, weightbearing, walking, stairs, squatting, running, jumping, prolonged sitting with flexed knees, pain, swelling, painful patellar movements, thigh muscle atrophy, and flexion deficiency. Participants selected a single response for each of the 13 categories which best described their knee pain. All 13 categories were summated to provide a final score, where 0 represented maximal disability and 100 represented no disability. The AKPS has established reliability and validity in PFP [32, 34, 35].
(iii) The Knee injury and Osteoarthritis Outcome Score (KOOS) [37] is a questionnaire that is recommended for use in studies of individuals with PFP and PFOA. The KOOS includes five subscales for pain, symptoms, function in activities of daily living, function in sport/recreation, and knee-related quality of life (QoL). The patellofemoral pain and osteoarthritis subscale (KOOS-PF) is an 11-item subscale used in conjunction with the original KOOS [33]. Each item contains a 5-point Likert scale, from zero (no knee problems) to four (extreme knee problems). A normalised score was calculated ranging from 0 to 100 (0 = extreme knee problems, 100 = no knee problems). The KOOS and KOOS-PF has established reliability, validity, and responsiveness in PFP and PFOA populations [33, 38].

### Statistical analyses

The participants’ most symptomatic knee (or in the case of bilateral, equally symptomatic knees, the right knee) was analysed. All data were explored for normality. Univariate associations between foot and ankle characteristics, and measures of knee symptoms and function were explored using correlation statistics for continuous measures (Pearson’s *r* for normally distributed data, and Spearman’s rho for non-normally distributed data). As both foot and ankle characteristics, and measures of knee symptoms and function were significantly associated with age, partial correlations adjusting for age were also calculated. The coefficient of determination (*R*^2^) was also calculated in order to ascertain how much the variation in knee symptoms and function were explained by the foot and ankle characteristics. Correlations were interpreted as small (0.1 to 0.3), moderate (0.3 to 0.5), large (0.5 to 0.7), and very large (0.7 to 0.9) [39], with significance set at *p* < 0.05. All statistical analyses were conducted using IBM SPSS Statistics Version 25 for Windows (IBM Corporation, NY, USA).

## Results

**Fig. 2:**
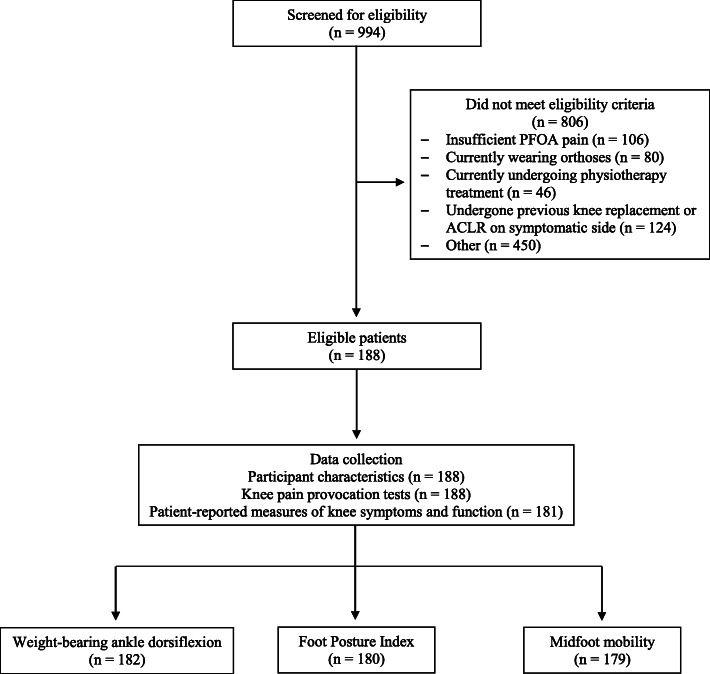
Participant flow through the study

Participant flow through the study is shown in Fig. 2. Nine hundred and ninety-four individuals volunteered for the RCT and 806 were excluded, leaving 188 for the total analysis. There were missing data for patient-reported measures of knee symptoms and function (*n* = 7), weightbearing ankle dorsiflexion range of motion (*n* = 4), FPI (*n* = 8), and midfoot mobility magnitude (*n* = 0). Participant characteristics are displayed in Table 1. Overall, there were 126 (67%) women, with a mean ± SD age of 59.9 ± 7.1 years, BMI of 29.3 ± 5.6 kg/m^2^, and duration of pain 7.0 ± 8.5 years. Mean weightbearing ankle dorsiflexion range of motion, FPI, and midfoot mobility magnitude values were 9.2 ± 6.7 cm, 3.5 (range: − 5 to 10), and 14.8 ± 4.9 mm, respectively.

**Table 1:** Participant characteristics. Values are mean (SD) unless otherwise stated

Participant characteristics	
Age, years	59.9 (7.1)
Number of females †	126 (67)
BMI (kg/m^2^)	29.3 (5.6)
Pain duration (years)	7.0 (8.5)
Study knee, left/right †	75 (39.9) / 113 (60.1)
History of knee injury (study limb) †	55 (29.3)
History of surgery (study limb) †	35 (18.6)
Foot and ankle characteristics	
WB ankle dorsiflexion ROM (study limb), cm	9.2 (6.7)
WB ankle dorsiflexion ROM (non-study limb), cm	10.1 (9.4)
FPI (study limb) ^	3.5 (−5 to 10)
FPI (non-study limb) ^	3.5 (−3 to 10)
Arch height difference (study limb), mm	12.8 (4.7)
Midfoot width difference (study limb), mm	6.3 (4.4)
Foot mobility magnitude (study limb), mm	14.8 (4.9)
Knee pain provocation tests	
Number of repeated single step-ups	8.6 (9.2)
Number of repeated double-leg sit-to-stand	9.3 (9.8)
Patient-reported measures of knee symptoms and function	
Average knee pain, mm	41.1 (22.1)
Worst knee pain, mm	55.7 (25.9)
Maximum knee pain during stair ambulation, mm	54.6 (25.0)
Maximum knee pain during squatting, mm	59.8 (27.4)
Maximum knee pain rising from sitting, mm	42.7 (25.3)
AKPS	59.7 (14.1)
KOOS-symptoms	45.3 (20.2)
KOOS-pain	44.7 (19.0)
KOOS-ADL	42.7 (24.4)
KOOS-sport/rec	48.6 (23.9)
KOOS-QoL	47.2 (20.1)
KOOS-PF	46.0 (20.6)

† Data presented as n (%)

^ Data presented as n (range)

**WB:** weightbearing; ROM: range of motion; FPI: Foot Posture Index (supinated = 0 to -10; neutral = +1 to + 7; pronated = + 8 to +10) **mm:** millimetres (0 = no pain; 100 = worst pain possible); AKPS: Anterior Knee Pain Scale (0 = maximum disability; 100 = no disability) **KOOS:** Knee injury and Osteoarthritis Outcome Score (0 = extreme knee problems; 100 = no knee problems); **ADL:** activities of daily living; **sport/rec:** sport and recreation; **QoL:** quality of life; **PF:** patellofemoral

Three foot and ankle characteristics demonstrated small statistically significant correlations with pain provoking tasks and patient-reported measures of knee symptoms and function (Table 2 and Additional File 1). Lower weightbearing ankle dorsiflexion range of motion had a small significant association with higher average knee pain (partial *r* = − 0.272, *p* < 0.001), higher maximum knee pain during stair ambulation (partial *r* = − 0.164, *p* = 0.028), and lower scores on the AKPS (indicative of greater disability; partial *r* = 0.151, *p* = 0.042). A higher FPI score (indicative of a more pronated foot posture) was significantly associated with fewer repeated single step-ups (partial *r* = − 0.181, *p* = 0.023) and double-leg sit-to-stands to knee pain onset (partial *r* = − 0.202, *p* = 0.022), although the magnitude of the associations were small. Greater midfoot mobility magnitude had a small significant association with fewer repeated single step-ups (partial *r* = − 0.197, *p* = 0.009) and repeated double-leg sit-to-stands to knee pain onset (partial *r* = − 0.169, *p* = 0.045). Adjusting for age slightly attenuated these associations.

**Table 2:** Univariate statistical analyses of associations between foot and ankle characteristics with knee symptoms and function

	Weightbearing ankle dorsiflexion range of motion			Foot Posture Index (FPI)			Foot mobility								
							Arch Height Difference^			Midfoot Width Difference^			Foot Mobility Magnitude		
	Correlation	*R*^2^	*p* value	Correlation	*R*^2^	*p* value	Correlation	*R*^2^	*p* value	Correlation	*R*^2^	*p* value	Correlation	*R*^2^	*p* value
Knee pain provocation tests:															
Repeated single step-ups	0.136	0.018	0.070	− 0.183	− 0.033	0.021*	− 0.094	− 0.009	0.212	− 0.133	− 0.018	0.076	− 0.201	− 0.040	0.007*
Repeated double-leg sit-to-stand	0.125	0.016	0.135	−0.202	− 0.041	0.021*	− 0.045	− 0.002	0.591	− 0.096	− 0.009	0.267	− 0.170	− 0.029	0.043*
Patient-reported measures of knee symptoms and function:															
Average knee pain	−0.277	− 0.077	< 0.001*	− 0.059	− 0.003	0.461	0.036	0.001	0.628	−0.053	− 0.003	0.482	0.030	0.001	0.693
Worst knee pain	−0.140	−0.020	0.059	−0.035	− 0.001	0.664	0.037	0.001	0.625	−0.057	−0.003	0.444	0.019	0.000	0.799
Max knee pain during stair ambulation	−0.173	−0.030	0.020*	−0.007	− 0.000	0.932	0.008	0.000	0.920	0.047	0.002	0.534	0.040	0.002	0.596
Max knee pain during squatting	−0.059	−0.003	0.433	0.039	0.002	0.629	0.133	0.018	0.078	0.146	0.021	0.052	0.105	0.011	0.163
Max knee pain rising from sitting	−0.073	−0.005	0.331	0.002	0.000	0.979	0.068	0.005	0.366	0.001	0.000	0.988	0.064	0.004	0.398
AKPS	0.159	0.025	0.032*	−0.124	−0.015	0.118	−0.124	− 0.015	0.098	− 0.037	−0.001	0.626	−0.116	− 0.013	0.123
KOOS-symptoms	0.062	0.004	0.405	0.052	0.003	0.517	−0.045	− 0.002	0.547	0.108	0.012	0.150	−0.039	−0.002	0.603
KOOS-pain	0.074	0.005	0.319	0.093	0.009	0.241	−0.057	−0.003	0.449	0.077	0.006	0.304	−0.039	−0.002	0.600
KOOS-ADL	0.091	0.008	0.223	0.082	0.007	0.300	−0.095	−0.009	0.205	0.066	0.004	0.381	−0.091	−0.008	0.225
KOO-sport/rec	0.058	0.003	0.441	0.032	0.001	0.690	−0.011	−0.000	0.883	0.000	0.000	0.996	−0.018	−0.000	0.807
KOOS-QoL	0.084	0.007	0.262	0.078	0.006	0.326	0.042	0.002	0.572	0.003	0.000	0.964	0.019	0.000	0.802
KOOS-PF	0.048	0.002	0.522	0.026	0.001	0.746	0.042	0.002	0.579	−0.006	−0.000	0.941	0.009	0.000	0.905

*p ≤ 0.05

^ data not normally distributed and presented as Spearman’s Rho

**mm:** millimetres (0 mm = no pain; 100 mm = worst pain possible); **AKPS:** Anterior Knee Pain Scale (0 = maximal disability; 100 = no disability); **KOOS:** Knee injury and Osteoarthritis Outcome Score; (0 = extreme knee problems; 100 = no knee problems), **ADL:** activities of daily living; **sport/rec:** sport and recreation; **QoL:** quality of life; **PF:** patellofemoral

Arch height difference and midfoot width difference were not significantly associated with any of the knee pain provocation tests or patient-reported measures of knee symptoms and function, and no foot and ankle characteristics were significantly associated with the KOOS subscales (*p* > 0.05) (Table 2).

## Discussion

The objective of this study was to explore the relationship between weightbearing ankle dorsiflexion range of motion, foot posture, and midfoot mobility, with measures of knee symptoms and function in individuals with clinically diagnosed symptomatic PFOA. Results demonstrated that lower weightbearing ankle dorsiflexion range of motion was significantly associated with worse patient-reported knee pain and greater disability (average knee pain, maximum knee pain during stair ambulation, and AKPS score), whilst a higher FPI (indicative of a more pronated foot posture) and greater midfoot mobility magnitude (indicative of a more flexible midfoot) were both significantly associated with earlier pain onset during PF joint (PFJ) loading tasks (i.e. fewer repeated single step-ups and double-leg sit-to-stands to knee pain onset). Although the magnitude of these associations was small, these findings suggest that selected foot and ankle characteristics may, in part, influence knee pain severity and functional capacity in individuals with PFOA.

Lower weightbearing ankle dorsiflexion range of motion was significantly associated with higher self-reported pain for average knee pain, higher maximum knee pain during stair ambulation, and worse AKPS score (indicative of greater disability). These findings are similar to those of Wyndow and colleagues [20], who reported that a lower weightbearing ankle dorsiflexion range of motion was associated with a greater frontal plane projection angle – a measure of dynamic knee valgus during functional tasks (e.g. stair ambulation) [40], which may initiate knee symptoms in this population [4]. Therefore, it is possible that the relationship between lower weightbearing ankle dorsiflexion range of motion with greater knee pain severity in individuals with PFOA, is mediated by increases in dynamic knee valgus as a result of compensatory mechanisms around the hip [41, 42], during functional tasks such as stair ambulation.

Higher FPI (indicative of a more pronated foot posture) was significantly associated with fewer repeated single step-ups and double-leg sit-to-stands to knee pain onset. Foot pronation has previously been highlighted as being associated with PFP [13, 15] and may therefore influence the efficacy of in-shoe interventions in this population [43], with recent studies providing preliminary evidence that foot orthoses may be an effective treatment in individuals PFOA [44–46]. One of the proposed mechanisms is that foot pronation, leading to internal tibial and femoral rotation, causes either a decrease in PFJ contact area or an increase in PFJ reaction forces, thereby elevating PFJ loads [19]. Given that single step-ups and double-leg sit-to-stand repetitions are tasks associated with high PFJ loads [47–49], it is plausible that individuals with PFOA who exhibit a more pronated foot posture may experience earlier onset of pain or more severe pain during these tasks. This finding adds to the small body of evidence that a more pronated foot posture is associated with PFP [13, 15] and given the biomechanical similarities observed between PFP and PFOA [10], may also be associated with PFOA. However, given the magnitude of this association, this finding should be interpreted with caution.

Greater midfoot mobility magnitude (indicative of a more flexible midfoot) was also shown to be significantly associated with fewer single step-ups and double-leg sit-to-stand repetitions to knee pain onset. Similar to exhibiting a higher FPI, a possible explanation for this finding is that having a more mobile midfoot may cause internal tibial and femoral rotation, leading to an increase in PFJ load and therefore an earlier onset of pain when performing these tasks [19]. Alternatively, greater midfoot mobility may be a compensatory mechanism for the aforementioned lower weightbearing ankle dorsiflexion range, resulting in an increase in knee flexion in order to allow the body to move over the foot during these tasks, thereby initiating an earlier onset of pain. However, as we did not measure knee biomechanics in this study, further research is required in order to confirm this proposed mechanism.

This study has demonstrated that there is a small relationship between foot and ankle characteristics with knee pain and disability in individuals with symptomatic PFOA. This knowledge may assist in the development of multimodal interventions to improve knee symptoms and function in individuals with PFOA. For example, physical therapy interventions such as mobilisation with movement [50] may be able to increase weightbearing ankle dorsiflexion range of motion, and mechanical therapy interventions such as foot orthoses or footwear modifications may be able to improve foot function by supporting the arch (i.e. addressing pronated foot posture) and/or aiding in shock attenuation (i.e. addressing greater midfoot flexibility) [51]. This is supported by previous studies that have demonstrated that individuals with PFOA respond favourably to foot orthoses and/or footwear immediately [44, 52], and in the short-term (6 weeks) [46]. However, future trials are now required to determine if such interventions are effective in isolation versus being a part of a multimodal treatment plan.

A key strength of this study was that we recruited a large cohort of individuals with clinically diagnosed symptomatic PFOA. Prior studies have included participants with general knee pain [53–55], which has resulted in the inclusion of those with isolated TFOA or those whom may not be experiencing predominant PFOA pain. As such, the ability to generalise the results to a symptomatic PFOA population, who experience pain and physical limitations [4, 8], was limited. Furthermore, this study included foot and ankle measures that are reliable and easy to implement within clinical practice. However, this study needs to be viewed in light of four key limitations. Firstly, given the number of correlations conducted, it is likely that some of the correlations observed are chance findings. Secondly, the magnitude of the observed associations in our study were small, so the clinical significance of our findings needs to be interpreted with caution. Thirdly, there are likely to be other variables not measured in this study, both mechanical and non-mechanical, which may also demonstrate a relationship with knee symptoms and function in those with PFOA. Lastly, as this is a cross-sectional study, we are unable to confirm that the associations between the foot and ankle characteristics and knee symptoms and function are causal.

## Conclusion

Individuals aged 50 years and above with a clinical diagnosis of symptomatic PFOA who present with lower weightbearing ankle dorsiflexion range of motion report more severe knee pain and greater disability, whilst those who present with a more pronated foot posture or with greater midfoot mobility report an earlier onset of pain during single step-up and double-leg sit-to-stand tasks. Given the small magnitude of these relationships, it is unlikely that interventions aimed solely at addressing foot and ankle mobility will have substantial effects on knee symptoms and function in this population, but further studies are needed to confirm this.

## Supplementary information

**Additional file 1.**

## Data Availability

The datasets used and/or analysed during the current study are available from the corresponding author on reasonable request.

## Abbreviations

PFP: Patellofemoral pain
PFOA: Patellofemoral osteoarthritis
FPI: Foot Posture Index
ROM: Range of motion
RCT: Randomised controlled trial
BMI: Body mass indices
VAS: Visual analogue scale
AKPS: Anterior Knee Pain Scale
KOOS: Knee injury and Osteoarthritis Outcome Score
SD: Standard deviation
mm: millimetre
cm: centimetres
CI: Confidence interval
