# Supplementary material for: Associations of foot and ankle characteristics with knee symptoms and function in individuals with patellofemoral osteoarthritis
Source: J Foot Ankle Res. 2020 Sep 23;13:57. doi: 10.1186/s13047-020-00426-8 (PMC7509922; doi:10.1186/s13047-020-00426-8)
Supplement: Supplementary file 1 — Additional file 1. [file 13047_2020_426_MOESM1_ESM.docx]

| **Additional file 1.** Univariate statistical analyses of associations between foot and ankle characteristics with knee symptoms and function (partial correlations adjusted for age) | | | | | | | | | | | | | | | | |
| --- | --- | --- | --- | --- | --- | --- | --- | --- | --- | --- | --- | --- | --- | --- | --- | --- |
|  | Weightbearing ankle dorsiflexion range of motion | | | Foot Posture Index  (FPI) | | | | Foot mobility | | | | | | | | |
|  |  | | |  | | | | Arch Height Difference^ | | | Midfoot Width Difference^ | | | Foot Mobility Magnitude | | |
|  | Correlation | *R*^2^ | *p* value | Correlation | | *R*^2^ | *p* value | Correlation | *R*^2^ | *p* value | Correlation | *R*^2^ | *p* value | Correlation | *R*^2^ | *p* value |
| Knee pain provocation tests: | | | | | | | | | | | | | | | | |
| Repeated single step-ups | 0.141 | 0.020 | 0.059 | -0.181 | -0.033 | | **0.023*** | -0.095 | -0.009 | 0.207 | -0.133 | -0.018 | 0.077 | -0.197 | -0.039 | **0.009*** |
| Repeated double-leg sit-to-stand | 0.128 | 0.016 | 0.128 | -0.202 | -0.041 | | **0.022*** | -0.051 | -0.003 | 0.549 | -0.092 | -0.008 | 0.275 | -0.169 | -0.029 | **0.045*** |
| Patient-reported measures of knee symptoms and function: | | | | | | | | | | | | | | | | |
| Average knee pain | -0.272 | -0.074 | **<0.001*** | -0.056 | -0.003 | | 0.483 | 0.040 | 0.002 | 0.594 | -0.053 | -0.003 | 0.478 | 0.041 | 0.002 | 0.589 |
| Worst knee pain | -0.133 | -0.018 | 0.074 | -0.033 | -0.001 | | 0.680 | 0.041 | 0.002 | 0.581 | -0.058 | -0.003 | 0.438 | 0.028 | 0.001 | 0.715 |
| Max knee pain during  stair ambulation | -0.164 | -0.027 | **0.028*** | -0.004 | -0.000 | | 0.958 | 0.016 | 0.000 | 0.836 | 0.046 | 0.002 | 0.543 | 0.053 | 0.003 | 0.480 |
| Max knee pain during  squatting | -0.055 | -0.003 | 0.464 | 0.039 | 0.002 | | 0.630 | 0.140 | 0.020 | 0.063 | 0.146 | 0.021 | 0.053 | 0.111 | 0.012 | 0.114 |
| Max knee pain rising from sitting | -0.069 | -0.005 | 0.357 | 0.002 | 0.000 | | 0.981 | 0.072 | 0.005 | 0.341 | 0.001 | 0.000 | 0.993 | 0.068 | 0.005 | 0.368 |
| AKPS | 0.151 | 0.022 | **0.042*** | -0.128 | -0.016 | | 0.108 | -0.132 | -0.017 | 0.078 | -0.035 | -0.001 | 0.639 | -0.128 | -0.016 | 0.089 |
| KOOS-symptoms | 0.079 | 0.006 | 0.287 | 0.057 | 0.003 | | 0.479 | -0.032 | -0.001 | 0.671 | 0.106 | 0.011 | 0.157 | -0.021 | -0.000 | 0.779 |
| KOOS-pain | 0.090 | 0.008 | 0.228 | 0.097 | 0.009 | | 0.226 | -0.046 | -0.002 | 0.538 | 0.076 | 0.006 | 0.315 | -0.023 | -0.001 | 0.761 |
| KOOS-ADL | 0.112 | 0.013 | 0.133 | 0.087 | 0.008 | | 0.276 | -0.080 | -0.006 | 0.288 | 0.064 | 0.004 | 0.397 | -0.070 | -0.005 | 0.356 |
| KOO-sport/rec | 0.045 | 0.002 | 0.552 | 0.026 | 0.001 | | 0.747 | -0.024 | -0.001 | 0.753 | 0.003 | 0.000 | 0.964 | -0.039 | -0.002 | 0.609 |
| KOOS-QoL | 0.067 | 0.004 | 0.367 | 0.074 | 0.005 | | 0.355 | 0.028 | 0.001 | 0.709 | 0.006 | 0.000 | 0.937 | -0.003 | -0.000 | 0.968 |
| KOOS-PF | 0.037 | 0.001 | 0.621 | 0.022 | 0.000 | | 0.781 | 0.030 | 0.001 | 0.685 | -0.004 | -0.000 | 0.960 | -0.006 | -0.000 | 0.941 |
| ***p ≤ 0.05**  ^ data not normally distributed and presented as Spearman’s Rho  **mm:** millimetres (0mm = no pain; 100mm = worst pain possible); **AKPS:** Anterior Knee Pain Scale (0 = maximal disability; 100 = no disability); **KOOS:** Knee injury and Osteoarthritis Outcome Score; (0 = extreme knee problems; 100 = no knee problems), **ADL:** activities of daily living; **sport/rec:** sport and recreation; **QoL:** quality of life; **PF:** patellofemoral. | | | | | | | | | | | | | | | | |
